# Consumption of Big Game Remains by Scavengers: A Potential Risk as Regards Disease Transmission in Central Spain

**DOI:** 10.3389/fvets.2018.00004

**Published:** 2018-03-02

**Authors:** Ricardo Carrasco-Garcia, Patricia Barroso, Javier Perez-Olivares, Vidal Montoro, Joaquín Vicente

**Affiliations:** ^1^SaBio group, Instituto de Investigación en Recursos Cinegéticos (CSIC-UCLM-JCCM), Ciudad Real, Spain

**Keywords:** African swine fever, hunting remains, red fox, scavenging, tuberculosis, vulture, wild boar

## Abstract

Understanding the role that facultative scavenger species may play in spreading infectious pathogens, and even becoming reservoirs for humans, domestic and wild ungulates or, on the contrary, preventing the spread of disease, requires a prior understanding of the pattern of carrion scavenging in specific scenarios. The objectives of this paper are (i) to describe the guild of vertebrate scavengers and (ii) to study the species-specific, habitat, and management-related factors involved in the usage of gut piles in South Central Spain (SCS), a tuberculosis (TB) endemic area. We used camera trapping at 18 hunting piles on seven hunting estates. A total of eight bird and five mammal taxa were detected at the remains of hunting piles. The most frequently detected species in terms of number of gut piles visited (78%) and scavenged (61%) was the red fox *Vulpes vulpes*, followed by the griffon vulture *Gyps fulvus* (56% as regards both presence and scavenging) and the raven *Corvus corax* (61 and 39% as regards presence and scavenging, respectively). We evidenced that griffon vultures accounted for most of the scavenging activity in open habitats, while facultative mammal scavengers, red fox, and wild boar *Sus scrofa* made the highest contribution to scavenging in vegetation-covered habitats. In the case of wild boar, the gut piles deposited during the evening and night favored higher rates of scavenging, while the opposite pattern was observed for griffons. Overall, our findings suggest that when disposing of hunting remains in areas of risk as regards disease transmission it is particularly important to consider the access that facultative mammals, and especially wild boar, have to material, while the presence of the resource needs to be safeguarded to protect specialist scavengers of conservation value. These results are of particular relevance in the case of wild boar in the current context of re-emerging TB and emerging African swine fever (ASF) in Europe.

## Introduction

Hunting remains represent an essential food resource for the scavenger community and can play an important role in ecosystem diversity and community structure (1), but also in disease transmission (2–4). Animal by-products can spread diseases (e.g., virus, bacteria, parasites, or prions) or chemical contaminants (e.g., dioxins), and can be dangerous for animal and human health if not properly disposed of. A large number of infectious agents have been found in big game species, and usually, the most abundant big game species [e.g., wild boar or red deer in most Europe (5)] in a particular region are of the greatest concern as the risk of exposure by these animal remains may be the highest. There are some 20 species within Europe (Cervidae, Bovidae, Ovidae, and Suidae) adding up to 15 million and representing a standing biomass of more than 0.75 billion kg (6). For some pathogens, there are experimental and empirical evidence on the potential role of exposure to carcasses in disease spread (7, 8), although, to the best of our knowledge, no specific research addresses the importance of animal by-product generated during hunting activities. In spite of regulations, logistic and economic constraints often lead hunting remains to be left in the field, thus making them available for all scavenger species.

Scavengers participates in disease dynamics because they can be competent hosts to pathogens acquired through scavenging of infected material, they act as potential vectors for a number of diseases, or facilitators of disease spread once they open the carcass, contaminating the vicinity, and enabling the action of vectors [e.g., Ref. (7)]. For a wide variety of pathogens (often shared between livestock and wildlife) horizontal transmission *via* scavenging (or facilitated by) of contaminated material is possible, but the consequences for disease dynamics are unknown in most cases [e.g., Ref. (8, 9)]. For example, tuberculosis (TB, caused by the *Mycobacterium tuberculosis* Complex) transmission may increase when scavengers of infected tissues, such as carnivores or wild boar, become infected [e.g., Ref. (10, 11)]. Experimental research has also determined susceptibility of scavenger species to particular infections and the likelihood of pathogen passage through the gastrointestinal tract. The digestive system of carrion birds presents conditions of extreme acidity, around pH 1–1.2 in the stomach (12, 13). Not only specialist scavengers but also facultative scavengers, such as terrestrial carnivorous and omnivorous species, may consume big game remains [e.g., Ref. (14)]. It has been reported (8) the ability of chronic wasting disease (CWD) -infected brain material to pass through the gastrointestinal tract of coyotes (*Canis latrans*) following oral ingestion, and be infectious, demonstrating that mammalian scavengers could contribute to the translocation and contamination of CWD in the environment. Outbreaks of Aujeszky virus disease have been associated with cannibalism in wild boar [*Sus scrofa* (15)]. To exemplify a more recent concern, the consumption of and behavior toward the carcasses of con-specifics may potentially be involved in the emergent transmission of African swine fever [ASF (16)] in wild boar in Europe.

The relevance of scavengers for pathogens to persist must be specifically assessed for each system and epidemiological context. A recent worldwide study reviewing the scavenging frequency of vertebrate species recorded at hunting remains (14) indicated that in both Mediterranean and Temperate (North) Spain, the top three positions were occupied by wild boar (*S. scrofa*), red fox (*Vulpes vulpes*), and griffon vultures (*Gyps fulvus*). Nonetheless, no precise data are available in relation to the factors that may affect the relative contribution made by different species to the scavenging of hunting remains, which is essential if effective management is to be implemented. The present study was conducted in SCS (the province of Ciudad Real and its border with that of Toledo), in which wild ungulate population densities have greatly increased and an important commercial hunting industry has been developed in recent decades. In order to provide a basis on which to determine the ecological, conservation and sanitary relevance of hunting ungulate gut piles for the vertebrate scavenging community (specialized and facultative) in European Southwest Mediterranean areas, we aimed to (i) describe the guild of vertebrate scavengers and (ii) study the species-specific, carrion, and habitat related factors involved in the usage of gut piles.

## Materials and Methods

### Study Area and Field Procedures

**Figure 1:**
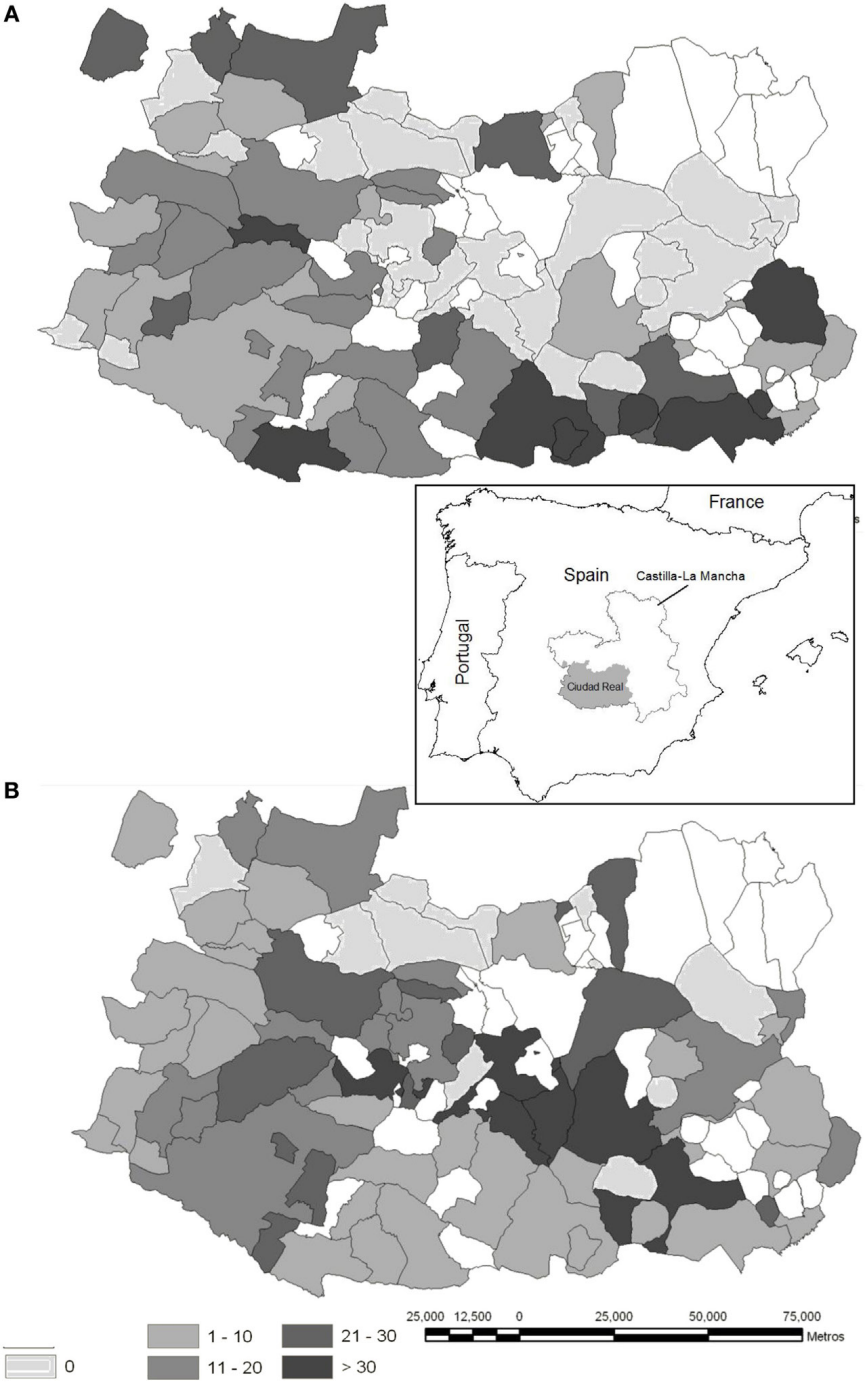
Maps illustrating the sampling sites (white dots). The capture effort (average yearly value of animals shot per hunting event and hunting estate) is represented, which equates to the individual big game offal generated for red deer **(A)** and wild boar **(B)** at municipality level, respectively (red deer hunted in 57% of the province area, wild boar in 73%) in the province of Ciudad Real (Autonomous Region of Castilla-La Mancha, South Central Spain, location is depicted in the inset). More details on the origin of the data can be seen in Vicente et al. (22).

Eighteen ungulate gut piles (originating from hunted eviscerated wild boar and red deer) were monitored on seven hunting estates in the province of Ciudad Real and its neighboring areas in Central Spain (see Table 1, 37°13′48′′N to 39°31′43′′ N in latitude; 2°25′54′′W to 6°34′06′′W in longitude) immediately after the hunting session (Figure 1). This is a hilly area (the altitude of the sampling sites ranges between 600 and 1,100 m a.s.l.), which consists of the Montes de Toledo and Sierra Morena mountain chains, which are connected by the Guadiana river valley, a fragmented agricultural and woodland habitat. This habitat is Mediterranean and is characterized by *Quercus ilex* forests and scrublands (dominated by *Cystus* spp., *Pistacia* spp., *Rosmarinus* spp., *Erica* spp., and *Phyllirea* spp.) with scattered pastures and small areas of crops. The hunting estates studied are fenced and devoted exclusively to ungulate hunting. This area has a rich biodiversity and includes an important population of monk vultures *Aegypius monachus* [over 170 breeding couples (17)] and griffon vultures (about 200 breeding couples in the provinces of Ciudad Real and Toledo). The Imperial eagle *Aquila adalberti* and golden eagle *Aquila chrysaetos* are also present. The principal ungulates are red deer (*Cervus elaphus*) and wild boar, but a low density of roe deer *Capreolus capreolus* is also present. The density of red deer in our study area ranges from approximately 0.10 to 0.40 ind/ha (18), and is usually higher than that of wild boar (19). A more detailed description of the study province can be seen in Vicente et al. (20).

**Table 1:** General descriptors concerning the monitoring and the usage of gut piles and mean values (±SD) for different habitats.

Gut pile	No. of ungulate hunted	Species	Habitat	Monitoring length per gut pile (days)	Days before first activity	Activity period per gut pile (days)	No. of pictures per camera
1	10	Red deer	Woodland	6.79	0.44	5.81	158
2	8	Red deer	Woodland	18.56	13.38	5.18	288
3	3	Wild boar	Woodland	16.54	7.93	8.52	72
4	3	Red deer	Open	7.46	2.86	3.81	58
5	2	Wild boar	Woodland	14.62	1.43	13.19	29
6	2	Red deer	Open	7.57	0.95	5.11	103
7	2	Red deer	Woodland	5.69	2.28	3.32	109
8	40	Red deer	Open	14.17	0.65	12.11	1
9	20	Both	Open	11.15	0.51	9.81	1,528
10	30	Both	Open	9.56	0.61	8.76	619
11	7	Both	Open	3.26	0.01	3.25	258
12	10	Both	Open	23.94	4.56	19.01	537
13	15	Both	Open	19.77	1.20	17.98	8
14	3	Both	Open	35.97	2.77	30.71	897
15	20	Both	Open	15.94	4.90	11.04	1,482
16	20	Both	Open	18.24	0.94	16.87	1,363
17	30	Wild boar	Open	4.65	0.35	4.13	657
18	2	Both	Woodland	31.65	0.65	27.86	1,125

		Mean values in open (*N* = 12)		14.31 ± 9.29	1.69 ± 1.67	11.88 ± 8.13	625.92 ± 578.09

		Mean values in woodland (*N* = 6)		15.64 ± 9.42	4.35 ± 5.22	10.65 ± 9.1	296.83 ± 415.4

*Activity period per gut pile (days) is defined as the period of time occurring from the first activity until the last scavenging activity detected in the gut pile*.

We used big game remains generated after big hunting activities between November and February in the years 2008 and 2009, which principally constituted thoracic and abdominal viscera, together with some non-trophy heads and hoofs. The piles of hunting remains were monitored by means of automatic digital camera traps. The cameras were the Leaf River IR-3BU model (Leaf River Outdoor Products, Taylorsville, MI, USA), a 4-megapixel passive system. Each camera was carefully placed at an approximate distance of 3 m from the gut piles, and between 50 and 90 cm above ground using wood poles or natural vegetation.

We visually considered two different types of habitats, since scavenger presence and carrion detection may vary: open (dehesas, i.e., savannah-like land, pastures, sown fields) and covered (woodlands, brushwoods, forests). The size of the habitat patch on which cameras were set was greater than 5 ha, which was assessed by QGis (21). The gut piles and cameras were checked every 2 days. The gut piles were visited until total consumption (only fur and bones completely cleaned of flesh remained), and the cameras were only removed 3 days after scavenging activity was detected for the last time. Table 1 indicates the number of animals from which the remains originated, the species, the habitat in which the hunting remains were placed, and the period of time during which the hunting remains were monitored. TB is present on all the study sites, which were the subject of a study by Vicente et al. (2011, 2013), although the specific prevalence in the study remains was not assessed.

### Data Analysis

The pictures were downloaded as JPEG files, visualized, and interpreted. Each picture is an individual case. Information was linked to each individual picture, including the date and time of capture, the scavenger species present, the number of individuals, and the type of scavenging behavior. We interpreted that scavenging takes place when a clear attitude of consumption of the hunting remains is detected, which is demonstrated by the oral physical contact of the animal with the hunting remains, swallowing and/or chewing. The general descriptors concerning the monitoring and the usage of hunting remains and the scavenging community were calculated for each pile, after which we calculated the average values per pile.

Inferential statistics was applied to explore the contribution of each species to the total presence and scavenging behavior as response variables, respectively. We used Generalized Linear Mixed Models (GLMMs) separately for each of the four main species detected (griffon vulture, raven *Corvus corax*, wild boar, and red fox), in which the number of pictures for a given scavenger and pile relative to the total number of pictures collected in each pile was a case (binomial response variable). We performed separate models for presence and scavenging behaviors, respectively. The type of habitat (open or close, as categorical) and the period of the day during which the big game remains were placed (categorical binomial; morning from 07:00 a.m. to 18:00 p.m.; evening/night from 18:00 p.m. to 07:00 a.m.) were considered as explanatory variables. We used a binomial error and a logit-link function. The location (estate) was included as a random factor. All the analyses were carried out using SAS (Glimmix Procedure; SAS version 9.1.3. SAS Institute Inc., Cary, NC, USA). The level of significance was established as <5%.

## Results

The general descriptors concerning the monitoring and the usage of hunting piles are shown in Table 1. Overall, the mean (±SD) period of monitoring per pile (time between the beginning and the end of the monitoring) was 14.75 ± 9.08 days. The mean period before the first activity (time between the start of the carrion monitoring and the first activity detected) was 2.58 ± 3.39 days. The mean period of activity per gut pile (time between the first and last activities detected) was 11.47 ± 8.22 days. Scavenging activity was detected at all the study hunting piles except one. The mean number of pictures per camera was 516 ± 541.

A total of eight bird and five mammal taxa were detected by the camera traps around the hunting remains (Table 2). In terms of the total number of pictures, the griffon vulture was the most frequently detected species, followed by ravens, monk vultures, azure-winged magpies *Cyanopica cyanus*, magpies *Pica pica*, wild boar, red fox, and red deer. Domestic dogs, usually hunting dogs that could not be retrieved by the hunters immediately after the hunting day, were found in 44 pictures (four gut piles). The Imperial eagle was recorded in only 19 pictures (two piles), while the Egyptian vulture *Neophron percnocterus*, Golden eagle, and common genet *Genetta genetta* were detected in 4, 4, and 1 pictures, respectively (in one pile). With regard to wild boar, we recorded activity in 17% of all 207 camera-nights (during the period of activity), and two or more visits were made by this species in 5% of these cases.

**Table 2:** Scavenging community and general parameters of activity detected.

Species detected	No. of gut piles visited		No. of gut piles scavenged		Proportion of days with presence (%)^a^	Proportion of days with presence (% open/woodland)^b^	No. of gut piles discovered	No. of arrived at second	No. of pictures^c^	Maximum group size	Mean group size (±SD)
	open	woodland	open	woodland							
Griffon vulture	9	1	9	1	22.3 (11.6)	26.4/2.8	1	5	4,026	120	36.0 ± 22.7
Monk vulture	7	0	7	0	18.4 (7.8)	22.3/0	0	0	2,800	15	4.2 ± 2.9
Raven	8	2	6	1	27.6 (17.0)	29.9/16.7	1	4	3,651	35	4.3 ± 3.5
Magpie	2	0	2	0	6.3 (3.4)	7.6/0	2	0	1,308	44	6.1 ± 4.1
Azure-winged magpie	5	1	5	1	28.6 (16.5)	34.6/0	5	0	1,419	22	4.2 ± 3.1
Egyptian vulture	1	0	0	0	1.0 (0)	1.2/0	0	0	4	1	1
Imperial eagle	2	0	2	0	1.0 (0)	1.2/0	1	1	19	2	1.3 ± 0.5
Golden eagle	1	0	1	0	1.0 (0)	1.2/0	0	1	4	1	1
Wild boar	4	4	4	3	17.0 (4.8)	12.9/36.1	3	1	437	6	1.6 ± 0.9
Red fox	9	5	7	4	32.0 (16.5)	31.1/38.9	3	2	416	2	1.0 ± 0.1
Common genet	0	1	0	0	0.5 (0)	0.6/0	0	0	1	1	1
Dog	3	1	3	1	3.9 (0.5)	3.5/5.6	2	1	44	2	1.0 ± 0.2
Red deer	2	0	2	0	6.8 (3.9)	8.2/0	0	0	179	1	1

*^a^The total effort consisted of 206 camera trap days. The figures in parentheses indicate the proportion of camera trap days on which more than one visit by the respective species was recorded*.

*^b^The total effort consisted of 170 and 36 camera trap days for open and woodland habitats, respectively*.

*^c^The total number of pictures in the study was 9,094 (note that in some cases, various species can be detected in the same pictures)*.

The species most frequently discovered at the gut piles (at five piles, four of which were in open areas) was the azure-winged magpie (which was also the first species detected at the pile after setting up the camera trap), followed by wild boar and red fox (three gut piles), magpies and domestic dogs (two gut piles), and finally griffon vultures, ravens, and Imperial eagles (one gut pile). Nonetheless, griffon vultures were the second species to arrive at the pile in five cases and ravens in four cases.

The griffon vultures, monk vultures, ravens, magpies, and azure-winged magpies accounted for most of the pictures and had the highest group sizes, especially the griffons (Table 2) which accounted for over 35 individuals, reaching a maximum group of around 120 individuals (compared, for instance, with that of the monk vulture of which there were no more than a maximum of 15). The average group size ranged between 4 and 6 for other birds, the most remarkable being that of ravens; the maximum group size reached almost 44 individuals. With regard to mammals, the average group size typically ranged between 1 and 2 for red fox and wild boar.

**Figure 2:**
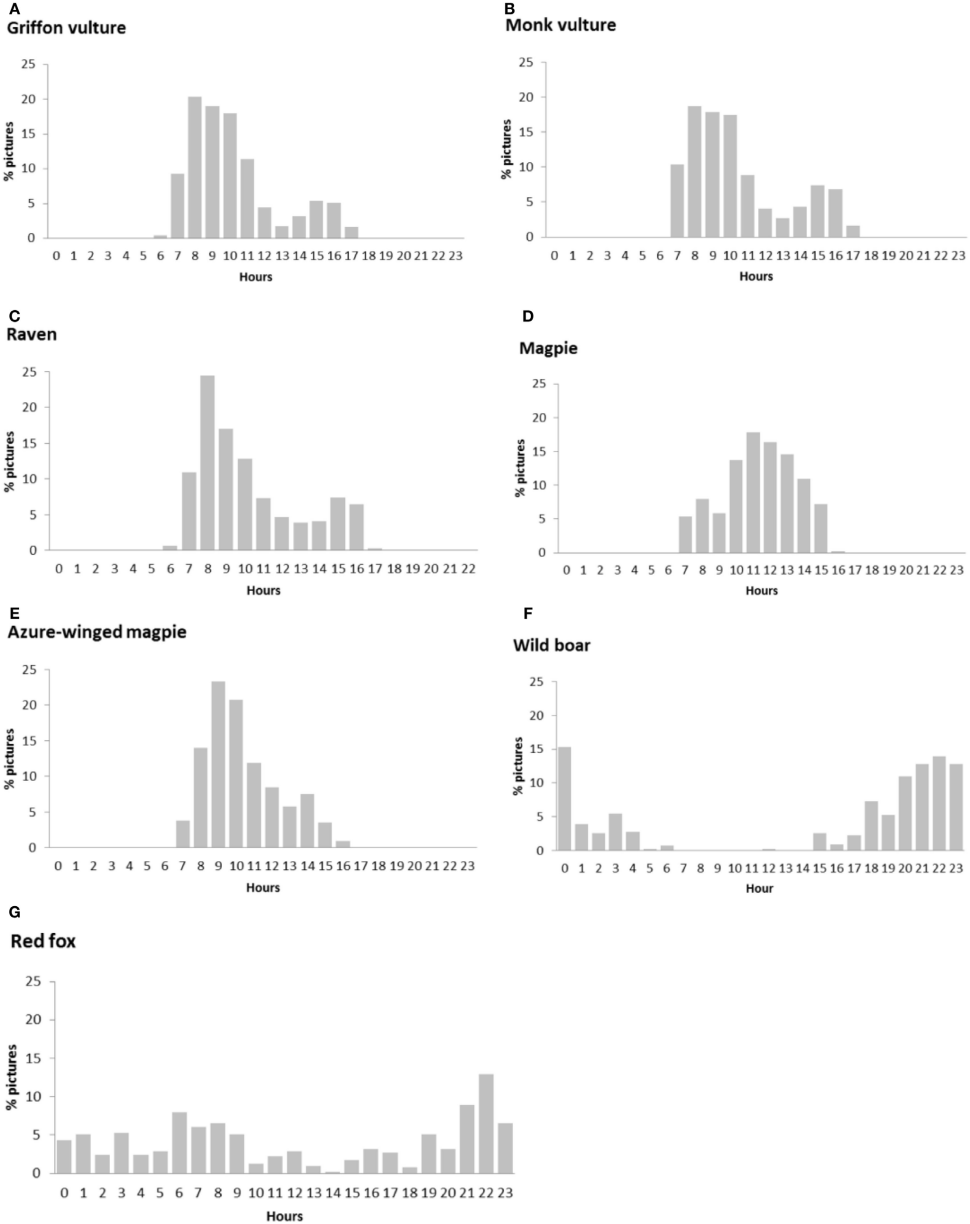
Circadian activity for the principal scavenger species calculated by means of picture frequency in each hour: **(A)** griffon vulture, **(B)** monk vulture, **(C)** raven, **(D)** magpie, **(E)** azure-winged magpie, **(F)** wild boar, and **(G)** red fox.

The daily activity of the main scavenger species, in terms of relative frequency of pictures per hour, is shown in Figure 2. Bird species were active during the daytime, but in the particular case of griffon vultures, monk vultures, and ravens, activity decreased in the central hours of the day. Wild boar and red fox had a nocturnal pattern, with activity peaking after sunset and at sunrise in the case of the wild boar, and certain continuous activity during the daytime in that of the red fox.

The percentage of gut piles, along with the presence and scavenging behavior of a given species, are shown in Table 3. The red fox was the most frequently detected species in terms of the number of gut piles visited (78%) and eventually scavenged (61%), followed by the griffon vulture (56% as regards both presence and scavenging) and the raven (61 and 39% as regards presence and scavenging, respectively). The monk vulture, wild boar, and azure-winged magpie had intermediate values of presence and scavenging (ranging from 31 to 45%), while the other species had lower values. It is important to mention that no scavenging was observed in the case of the common genet and the Egyptian vulture on the rare occasions on which they were detected. Interestingly, a solitary male red deer was detected scavenging two piles (feeding on gut contents) on the same hunting estate during 10 different nights in January and late February.

**Table 3:** Proportion (%) of gut piles detected or scavenged by species (*) and average contribution to presence (% of pictures with presence) and scavenging activity detected (% of picture in which scavenging was detected) per species and pile.

Species	Total (*N* = 18)		Open (*N* = 12)	Woodland (*N* = 6)
	Pres/Scav^a^	%Pres/%Scav^b^	%Pres/%Scav^b^	%Pres/%Scav^b^
Griffon vulture	55.6/55.6	30.2/30.3	45.2/46.7	0.1/0.3
Monk vulture	38.9/38.9	24.1/13.5	36.2/20.8	0/0
Raven	61.1/38.8	11.8/14.3	15.7/18.6	4.9/6.0
Magpie	11.1/11.1	6.4/6.7	9.7/10.3	0/0
Azure-winged magpie	33.3/33.3	16.5/24.1	21.4/29.1	6.8/15.1
Egyptian vulture	5.6/0	<0.0/0	0.02/0	0/0
Imperial eagle	11.1/11.1	0.5/0.2	0.7/0.31	0/0
Golden eagle	5.6/5.6	<0.1/0.01	0.05/<0.1	0/0
Wild boar	44.4/38.9	13.0/14.1	1.8/1.9	35.5/36.5
Red fox	77.8/61.1	15.5/12.5	12.0/2.5	22.6/30.8
Common genet	5.6/0.0	<0.0/0	0/0	0.01/0
Dog	22.2/22.2	4.7/8.6	3.3/7.1	7.3/11.35
Red deer	11.1/11.1	1.3/0.6	2.0/0.9	0/0

*^a^Percentage of gut piles at which the species was detected/percentage of gut piles at which scavenging by the species was detected*.

*^b^Average contribution to presence and scavenging behavior (% of pictures. *n* = 17 gut piles, since the gut pile at which scavenging was absent was excluded) for each species. This was calculated as the average no. of pictures in which a given species was detected (presence or scavenging, respectively) relative to the total number of pictures per species and gut pile (presence or scavenging, respectively)*.

Both azure-winged magpies and Eurasian magpies (which usually weigh less than 100 and 200 weight in Spain, respectively) had particularly high presence and scavenging values in open habitats (Table 3). In order to make comparisons between species, we shall now consider the four main species (griffon vulture, red fox, wild boar, and raven) on the basis or their detected contribution to scavenging, size and quantitative capacity for scavenging. In terms of the average proportion of pictures per gut pile, griffons accounted for most of the presence and scavenging activities in open habitats, followed by ravens. Red fox and wild boar had lower values, although in close habitats, they made the highest average contributions to presence and scavenging, followed by ravens, while vultures had lower values (very low compared with open habitats).

**Figure 3:**
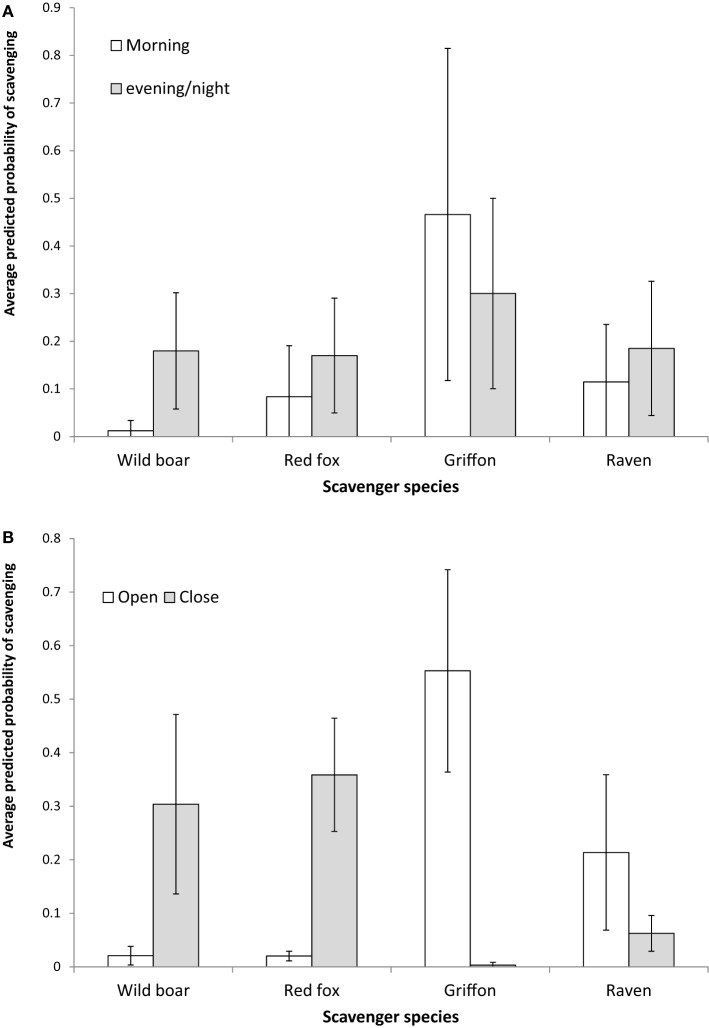
Average predicted probability of scavenging (and 95% CI SE) from the Generalized Linear Mixed Models for the contribution made by scavenging to the total per pile for the main species as a function of the habitat **(A,B)** the period of day during which the hunting remains were deposited. Please note that these values were “corrected” by other variables incorporated into the model, such as the estate.

The results obtained as regards the statistics concerning the models for the relative contribution of each species to presence and scavenging behavior, respectively (Table 4) were similar. The griffon’s contribution to presence and scavenging activity was statistically higher in open habitats, whereas for wild boar and red fox, it was higher in close habitats (Figure 3). No statistical effect of habitat was detected for ravens. Wild boar statistically contributed more to presence and scavenging when gut piles were deposited during the evening/night period, while griffons and ravens had statistically higher rates of presence and scavenging when piles were deposited during the morning (Figure 3). No statistical effect of the period of time was evidenced for the red fox.

**Table 4:** The statistics concerning the models for the relative contribution of each species to the total presence and to the total scavenging, respectively (no. of pictures/total no. of pictures), per pile.

Effect 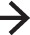	Habitat		Period of carrion placement	
**Presence models**				
Wild boar	Open = −4.77 ± 0.8431.75	**<0.01**	Morning = −2.70 ± 0.4535.50	**<0.01**
Red fox	Open = −2.56 ± 0.6625.02	**<0.01**	Morning = 0.10 ± 0.1930.27	0.63
Griffon	Open = 9.89 ± 1.1475.19	**<0.01**	Morning = 5.99 ± 0.23638.99	**<0.01**
Raven	Open = −0.05 ± 0.540.01	0.92	Morning = 1.35 ± 0.12133.97	**<0.01**

**Scavenging models**				
Wild boar	Open = −5.09 ± 1.1619.11	**<0.01**	Morning = −2.38 ± 0.4626.45	**<0.01**
Red fox	Open = −2.74 ± 0.4242.61	**<0.01**	Morning = −0.33 ± 0.241.88	0.19
Griffon	Open = 9.73 ± 1.1374.02	**<0.01**	Morning = 6.38 ± 0.28532.19	**<0.01**
Raven	Open = −3.39 ± 1.7703.53; 1,14	0.08	Morning = 0.86 ± 0.1249.10; 1,14	**<0.01**

*The reference values for parameter estimates (±SE) were for the level “evening-night” in the variable “period of time,” and for the level “woodland” in the variable “habitat.” The degrees of freedom (d.f.) for the numerator and denominator were 1 and 15 for the presence models; and 1 and 14 for the scavenging models, respectively. The *F*-values are shown at the bottom of the cells*.

*Significant *p*-values (<0.05) are higlighted in bold*.

## Discussion

This research provides the first results on the factors that determine the usage of big game remains by scavengers in South Western Europe, a TB endemic region. The guild of vertebrates using big game remains in Mediterranean habitats in SCS appeared rich as regards the number of species, which supports the prevalence of facultative scavenging (14). Vultures and corvids were the most common diurnal scavenger at gut piles in the study area, especially in open habitats, and benefited from most of the piles available. While vultures, which are generally very resistant to ungulate infectious diseases, may contribute to the removal of most pathogenic microorganisms from dead animals (12, 23), the role of mammal scavengers such as wild boar and red fox requires further research. We evidenced that these mammal species prevailed at hunting piles located in covered areas (woodlands and scrublands), and that the moment of gut pile deposition influenced their subsequent usage. In the case of wild boar, those plies deposited during the evening and night favored scavenging by this species. Overall, our findings suggest that the disposal of hunting remains in areas of risk for disease transmission, and particularly TB in our study area, must particularly consider the access of facultative mammals, especially wild boar, to material, while the presence of the resource needs to be safeguarded in order to protect specialist scavengers of conservation value. We also raise concerns about the potential role of cannibalism by wild boar in relation to other pathogens not present in our study area, such as ASF (16).

The contribution made by azure-winged magpies and magpies to the consumption of hunting remains is limited, since these birds’ activity around the gut piles probably consisted of searching for decomposer insects, and their ingestion rate is very low when compared with that of larger birds. Vultures and ravens accounted for a relevant proportion of scavenging in open habitats, and particularly those which took place during the daytime. For instance, corvids were frequently involved in the early discovery of gut piles: they were detected 2.5 times faster in open habitats than in covered ones (1.6 and 4.3 days, respectively). The early consumption by birds determined the subsequent use of the hunting remains, and prevented the subsequent access of facultative mammal scavengers (usually nocturnal) once remains were left.

In woodlands, wild boar and red fox made the highest average contribution to scavenging (wild boar made 37% of the average contribution per species and gut pile, and red fox, 31%), whereas vultures were much less relevant (Table 3). From the ecological perspective, factors limiting vultures’ access to big game remains may have an effect on the ecology of facultative scavengers. For instance, the presence of vultures may reduce the scavenging opportunities of mesocarnivores (facultative scavengers, particularly red fox) through their indirect effect on abundance, as evidenced in two neighboring areas in South-eastern Spain (24).

Interestingly, in this study wild boar scavenged both cervid and wild boar guts, contrary to that which usually occurs with entire carcasses [the authors, unpublished (16)], when it (at least partially) avoids feeding on conspecifics. The red fox has been described as behaving in a similar manner (25). This highlights that feeding on conspecific gut piles, as compared with entire carcasses, probably involves an increased risk of pathogen transmission. In Mediterranean ecosystems the gut piles of big game may, therefore, be a source of inter and intraspecific transmission of pathogens, particularly in the case of wild boar.

The scavenging activity of wild boar should be considered as a risk factor to consider when applying control strategies for diseases such as TB and, eventually, ASF. This must be contextualized in integral disease–control approaches [e.g., the reduction of population abundance and aggregation, the management of risk factors, etc. (26)]. In central and East Europe, ASF virus infecting wild boar and pigs has a great ability to persist in the tissues of dead animals (27). As this viral disease becomes more chronic or carrier host status is possible, the importance of managing wild boar hunting remains and carcasses increases. It had been considered that carnivores, such as the endangered Iberian lynx, the badger and the red fox, may possibly have been infected by TB as a result of their consuming infected prey or carrion [e.g., Ref. (28, 29)] in South Central Spain, and this may similarly occur with carnivores in other latitudes [e.g., Ref. (30)]. Some relevant pathogens that may be transmitted *via* scavenging are the nematodes of the genus *Trichinella*, Aujeszky’s disease virus (from wild boar) or Hepatitis E virus. On the contrary, facultative scavengers, such as carnivores, are likely to reduce the intraspecific transmission risk of some pathogens, as in the case of brucellosis (31). We consider the presence of a male red deer at two gut piles as anecdotal. Dietary deficits and unhealthy conditions have been proposed as the origin of abnormal and stereotypic oral and diet behaviors in ungulates (32).

### Management Applications

The findings of this study recommend improving the previous detailed veterinary inspection of hunting remains in areas of risk as regards diseases (e.g., TB), followed by their appropriate disposal, preferably on mammal-proof sites (bird feeding stations) in order to deal with conservation issues, or the elimination by other authorized means when risk of disease spread is present. We evidenced that, in open localizations, avian scavengers are a suitable and ecological option for the removal of hunting remains. An interesting approach might be a combination of strategies: the use of open habitats, depositing hunting remains during the daytime, and implementing temporary fences that effectively limit access by mammals. Moreno-Opo et al. (33) have proposed cheap, mobile, and easily manageable enclosure models, such as electrified mesh, that prevent facultative mammalian scavengers from entering feeding stations, at least temporarily while remains are totally scavenged by specialists.

## Author Contributions

RC-G contributed to the field work, analyzed and interpreted the data, and drafted the manuscript. PB drafted the manuscript. JP-O contributed to the field work and interpreted the data. VM contributed to the design of the study and analyzed the data. JV contributed to the design of the study, analyzed the data, and drafted the manuscript.

## Conflict of Interest Statement

The authors declare that the research was conducted in the absence of any commercial or financial relationships that could be construed as a potential conflict of interest.

## References

[1] Selva N Fortuna MA. The nested structure of a scavenger community. Proc Biol Sci (2007) 274:1101–8. 10.1098/rspb.2006.0232 17301021 PMC2124470

[2] Corner LA. The role of wild animal populations in the epidemiology of tuberculosis in domestic animals: how to assess the risk. Vet Microbiol (2006) 112:303–12. 10.1016/j.vetmic.2005.11.015 16326039

[3] Jennelle CS Samuel MD Nolden CA Berkley EA Deer carcass decomposition and potential scavenger exposure to chronic wasting disease. J Wild Manage (2009) 73:655–62. 10.2193/2008-282

[4] Pozio E The opportunistic nature of *Trichinella*––exploitation of new geographies and habitats. Vet Parasitol (2013) 194:128–32. 10.1016/j.vetpar.2013.01.037 23433987

[5] Gortazar C Acevedo P Ruiz-Fons F Vicente J Disease risks and overabundance of game species. Eur J Wild Res (2006) 52:81–7. 10.1007/s10344-005-0022-2

[6] Apollonio M Andersen R Putman R Introduction. In: Apollonio M Putman R, editors. European Ungulates & their Management in the 21st Century. Cambridge: Cambridge University Press (2010). p. 1–11.

[7] Bellan SE Turnbull PC Beyer W Getz WM. Effects of experimental exclusion of scavengers from carcasses of anthrax-infected herbivores on *Bacillus anthracis* sporulation, survival, and distribution. Appl Environ Microbiol (2013) 79:3756–61. 10.1128/AEM.00181-13 23584788 PMC3675950

[8] Nichols TA Fischer JW Spraker TR Kong Q VerCauteren KC CWD prions remain infectious after passage through the digestive system of coyotes (*Canis latrans*). Prion (2015) 9:367–75. 10.1080/19336896.2015.1086061 26636258 PMC4964857

[9] Barron MC Tompkins DM Ramsey DSL Bosson MAJ. The role of multiple wildlife hosts in the persistence and spread of bovine tuberculosis in New Zealand. N Z Vet J (2015) 63:68–76. 10.1080/00480169.2014.968229 25384267 PMC4566902

[10] Ragg JR Mackintosh CG Moller H. The scavenging behaviour of ferrets (*Mustela furo*), feral cats (*Felis domesticus*), possums (*Trichosurus vulpecula*), hedgehogs (*Erinaceus europaeus*) and harrier hawks (*Circus approximans*) on pastoral farmland in New Zealand: implications for bovine tuberculosis transmission. N Z Vet J (2000) 48:166–75. 10.1080/00480169.2000.36188 16032148

[11] Renwick AR White PCL Bengis RG Bovine tuberculosis in African wildlife: a multi-species host–pathogen system. Epidemiol Infect (2007) 135:529–40. 10.1017/S0950268806007205 16959052 PMC2870607

[12] Houston DC Cooper JE The digestive tract of the whitebacked griffon vulture and its role in disease transmission among wild ungulates. J Wild Dis (1975) 11:306–13. 10.7589/0090-3558-11.3.306 239254

[13] Farner DS The hydrogen ion concentration in avian digestive tracts. Poultry Sci (1942) 21:441–5. 10.3382/ps.0210445

[14] Mateo-Tomas P Olea P Moleon M Vicente J Botella F Selva N From regional to global patterns in vertebrate scavenger communities subsidized by big game hunting. Divers Distrib (2015) 21:913–24. 10.1111/ddi.12330

[15] Gortazar C Vicente J Fierro Y Leon L Cubero MJ Gonzalez M Natural Aujeszky’s disease in a Spanish wild boar population. Ann N Y Acad Sci (2000) 969:210–2. 10.1111/j.1749-6632.2002.tb04380.x 12381593

[16] Probst C Globig A Knoll B Conraths FJ Depner K. Behaviour of free ranging wild boar towards their dead fellows: potential implications for the transmission of African swine fever. R Soc Open Sci (2017) 4:170054. 10.1098/rsos.170054 28573011 PMC5451812

[17] Jiménez J López-Izquierdo P Chapter: El buitre negro *Aegypius monachus* en el Parque Nacional de Cabañeros. In: Dobado PM Arenas R, editors. The Black Vulture: Status, Conservation and Studies. Ciudad Real y Toledo, Castilla-La Mancha, España: Consejería de Medio Ambiente de Andalucía (2012). p. 233–44.

[18] Acevedo P Ruiz-Fons F Vicente J Reyes-Garcia AR Alzaga V Gortazar C Estimating red deer abundance in a wide range of management situations in Mediterranean habitats. J Zool (2008) 276:37–47. 10.1111/j.1469-7998.2008.00464.x

[19] Acevedo P Vicente J Höfle U Cassinello J Ruiz-Fons F Gortazar C. Estimation of European wild boar relative abundance and aggregation: a novel method in epidemiological risk assessment. Epidemiol Infect (2007) 135:519–27. 10.1017/S0950268806007059 16893488 PMC2870594

[20] Vicente J Barasona JA Acevedo P Ruiz-Fons JF Boadella M Diez-Delgado I Temporal trend of tuberculosis in wild ungulates from Mediterranean Spain. Transbound Emerg Dis (2013) 60:92–103. 10.1111/tbed.12167 24171854

[21] The QGIS Development Team. *QGIS Geographic Information System* Open Source Geospatial Foundation (2009). Available from: https://qgis.org

[22] Vicente J Carrasco R Acevedo P Montoro V Gortazar C Big game waste production: sanitary and ecological implications. In: Kumar S, editor. Integrated Waste Management. Volume II. Rijeka, Croatia: Intech (2011). p. 97–128.

[23] Ogada DL Torchin ME Kinnaird MF Ezenwa VO. Effects of vulture declines on facultative scavengers and potential implications for mammalian disease transmission. Conserv Biol (2012) 26:453–60. 10.1111/j.1523-1739.2012.01827.x 22443166

[24] Morales-Reyes Z Sánchez-Zapata JA Sebastián-González E Botella F Martina Carrete M Moleón M Scavenging efficiency and red fox abundance in Mediterranean mountains with and without vultures. Acta Oecol (2017) 79:81–8. 10.1016/j.actao.2016.12.012

[25] Moleón M Martínez-Carrasco C Muellerklein OC Getz WM Muñoz-Lozano C Sánchez-Zapata JA. Carnivore carcasses are avoided by carnivores. J Anim Ecol (2017) 86:1179–91. 10.1111/1365-2656.12714 28609555

[26] Gortazar C Vicente J Boadella M Ballesteros C Galindo RC Garrido J Progress in the control of bovine tuberculosis in Spanish wildlife. Vet Microbiol (2011) 15:170–8. 10.1016/j.vetmic.2011.02.041 21440387

[27] Gavier-Widen D Gortazar C Stahl K Neimanis AS Rossi S Hard AV African swine fever in wild boar in Europe: a notable challenge. Vet Rec (2015) 176:199–200. 10.1136/vr.h699 25698823

[28] Millan J Jimenez MA Viota M Candela MG Peña L Leon-Vizcaino L. Disseminated bovine tuberculosis in a wild red fox (*Vulpes vulpes*) in southern Spain. J Wildl Dis (2008) 44:701–6. 10.7589/0090-3558-44.3.701 18689657

[29] Perez J Calzada J Leon-Vizcaino L Cubero MJ Velarde J Mozos E Tuberculosis in an Iberian lynx (*Lynx pardinus*). Vet Rec (2001) 148:414–5. 10.1136/vr.148.13.414 11327650

[30] Bruning-Fann CS Schmitt SM Fitzgerald SD Fierke JS Friedrich PD Kaneene JB Bovine tuberculosis in free-ranging carnivores from Michigan. J Wildl Dis (2001) 37:58–64. 10.7589/0090-3558-37.1.58 11272505

[31] Maichak EJ Scurlock BM Rogerson JD Meadows LL Barbknecht AE Edwards WH Effects of management, behavior, and scavenging on risk of brucellosis transmission in elk of western Wyoming. J Wild Dis (2009) 45:398–410. 10.7589/0090-3558-45.2.398 19395749

[32] Bergeron R Badnell-Waters AJ Lambton S Mason G Stereotypic oral behaviour in captive ungulates: foraging, diet and gastrointestinal function. 2nd ed In: Mason G Rushen J, editors. Stereotypic Animal Behaviour: Fundamentals and Applications to Welfare. Wallingford: Cab eBooks, CABI (2006). p. 325–53.

[33] Moreno-Opo R Margalida A Garcia F Arredondo A Rodriguez C Gonzalez LM Linking sanitary and ecological requirements in the management of avian scavengers: effectiveness of fencing against mammals in supplementary feeding sites. Biod Conserv (2012) 21:1673–85. 10.1007/s10531-012-0270-x

